# Supplementary material for: Are carbon emissions trading and green financial instruments synergistic? -Comprehensive quantitative research based on content analysis
Source: PLoS One. 2024 Mar 7;19(3):e0298601. doi: 10.1371/journal.pone.0298601 (PMC10919601; doi:10.1371/journal.pone.0298601)
Supplement: S1 Data — (ZIP) [file pone.0298601.s001.zip › Data/地级市Z_F工作报告.zip!/─┌├╔╣┼/░n═╖╩╨/└┤╫╘╣1⁄2╓┌║┼í╛┬φ┐╦╩2╛▌═°í┐_2019.docx]

各位代表：

现在，我代表市人民政府向大会报告工作，请予审议，并请市政协各位委员和列席人员提出意见。

一、2018年工作回顾

过去一年，我们在市委的坚强领导下，以习近平新时代中国特色社会主义思想为指导，深入学习贯彻党的十九大精神，全面贯彻落实习近平总书记对内蒙古重要讲话重要指示精神，统筹推进稳增长、促改革、调结构、惠民生、防风险各项工作，经济社会保持了健康稳定的发展态势。全市地区生产总值增长6.8%；社会消费品零售总额增长6.4%；一般公共预算收入增长6.2%；城乡居民收入分别达到47407元和17435元，分别增长7.2%和9.7%。

（一）经济运行稳中提质。全面落实国家、自治区稳增长的各项政策措施，为企业减轻各类税费负担50亿元，降低用电成本21.1亿元，争取各类政策资金100亿元，规模以上工业增加值增长11%，利润增长29.3%。协调金融机构支持包钢优化信贷结构、降低融资成本，包钢主要产品产量和营业收入创历史最高水平，利润增长近两倍。树立全市“一盘棋”理念，全力推动企地融合，制定出台《振兴实体经济促进企地融合若干政策措施意见》和配套方案，搭建产业协作、产品购销、信息通信、军民融合、科技创新、银企对接六大平台，原材料就地转化和产品本地购销比例大幅提升，31个企地融合项目取得积极进展。制定实施支持非公经济发展十条措施及配套政策，非公经济占全市地区生产总值、税收、科技创新成果、从业人员、企业数量的比重分别达到60%、73.6%、62%、75%和94%，4户优秀民营企业获得自治区表彰。全力稳定项目投资拉动，实施了681个亿元以上重点项目，完成投资1312.7亿元，300个项目陆续建成。引进国内（区外）到位资金225亿元，居自治区首位。

（二）产业结构加速优化升级。加快推动工业转型升级。制定实施传统工业转型升级三年行动方案，新恒丰铝电一体化、震雄铜拉丝等项目正式投产，包钢高强度稀土钢实现试生产，中科院金属研究所轴承钢冶炼、上海交大高纯氧化铝制备技术取得突破，北方股份国内首台混合动力矿用车成功下线，内蒙古网络协同制造云平台、北奔车联网平台获评国家工信部“2018年制造业双创平台试点示范项目”。制定实施战略性新兴产业发展三年行动方案，通威高纯晶硅、昊明镍氢动力电池等项目实现投产，光伏、新能源汽车等新产业链加速构筑，高新技术企业产值增长30%，战略性新兴产业占规模以上工业增加值比重达20%。制定实施稀土产业发展三年行动计划，出台支持稀土新材料产业园区发展十条政策，稀土新材料产业基地建设全面展开，中科精密伺服电机、江馨微电机等项目正式投产，希捷环保脱硝催化剂、中科世纪稀土硫化铈着色剂达到国际先进水平，中国稀土产业景气指数在包头发布，稀土功能材料及终端应用产品占比达到53.6%，“挖土卖土”粗放型资源开发模式持续改善。大力发展军民融合产业，投资20.3亿元的8个军民融合项目加快推进，成功举办第一届军民融合成果展和第十六期钱学森论坛。着力推动服务业提档升级。出台促进现代服务业高质量发展政策措施，服务业增加值占比达到55.8%。吾悦广场商业综合体开工建设，九原万达、铂尔曼酒店开业运营。现代物流“六园区三基地”完成交易额898亿元，传化交投公路港成为全国西北地区最大的零担货物集散中心，获评全国供应链创新与应用试点城市和生产服务型国家物流枢纽承载城市。自治区级工业设计中心数量全区第一，鹿王成为自治区唯一一个连续两次通过复核的国家级工业设计中心。渤海银行、金谷农商银行落户我市，17家企业挂牌上市。举办首届自治区旅游产业博览会、第五届中国·包头牛羊肉产业大会等66场大型综合展会，获评全国优秀会展城市。共享经济、电商平台等新业态新模式呈现良好发展势头，重点监控电商企业交易额完成1154亿元。旅游收入576亿元，增长14.7%，土右旗入选“2018年度中国十大全域旅游示范县市”，春坤山获批国家4A级景区。大力发展现代农牧业。新增特色作物19.3万亩，全年粮食产量达到109.5万吨，牲畜出栏612万头。建成76个标准化种养示范基地、15个现代农牧业科技示范基地、10个都市休闲观光农牧业示范基地，成功入选国家农业科技园区。新增市级龙头企业16家、市级示范合作社45家，新认证“三品一标”31个，小尾羊被裁定为中国驰名商标，肉菜流通二维码追溯体系经验向全国推广，粮食安全盟市长责任制考核连续两年居自治区首位。加速集聚科技创新要素。制定出台促进科技创新工作十条措施，新增国家级企业技术中心2家、院士工作站6家、自治区级重点实验室2个、工程中心2个、企业技术（研发）中心20个，实施关键共性技术攻关30项、科研项目转化13个，形成了一批具有自主知识产权的新技术、新工艺、新产品。中关村信息谷创新中心、清华启迪科技园落户包头。创立“中国稀土钢系列产品标准”等8项首创标准。科技研发经费投入强度居自治区首位。专利申请量突破3000件、增长19%，万人有效发明专利达到4.6件。制定出台加强重点领域人才工作十项措施，设立2亿元人才创新创业投资基金，新增高技能人才3614人，引进高层次和紧缺急需人才1036人，创新正在成为驱动高质量发展的新引擎。

（三）“三大攻坚战”开局良好。制定实施关于进一步强化政府债务化解工作意见，全面摸清政府债务及相应资产底数，千方百计化解政府债务214.9亿元，完成自治区考核目标任务的113.3%，“十个全覆盖”10项工程款30.78亿元全部化解。建立地方金融风险防范处置联席会议制度，强化金融风险摸排化解，金融机构不良贷款率3.29%，低于自治区1.21个百分点。制定实施脱贫攻坚三年行动方案，落实各类资金2.9亿元全力保障脱贫攻坚，全年实现稳定脱贫2945人，固阳具备贫困县摘帽条件。中央环保督察反馈的16个限期整改问题已整改完成13个，8个长期整改任务全部建立长效机制、持续推进。制定实施打赢蓝天保卫战三年行动计划等政策措施，实施97项环境污染治理工程，全面完成30万千瓦以上火电机组超低排放改造，通过清洁取暖改造、洁净燃料替代等方式减少原煤使用量10万吨，空气质量平均达标天数比例达到80.7%，提高3.4个百分点，PM2.5平均浓度下降到39微克每立方米，降低11.4个百分点。完成四道沙河、二道沙河河道疏浚和堤防加固工程，综合整治城区河道环境34.5公里，建成区黑臭水体基本消除。封停关闭超采区地下水源井64眼，全面开展饮用水水源地保护区规范化建设，黄河三断面和城市集中式饮用水水源地水质达标率100%。采取新建、租借、共用等方式完成8个工业园区固废渣场整改，实现固废全过程、闭环式管理。

（四）改革开放持续深化。出台“放管服”改革系列政策措施，全面实施证照分离改革，“多证合一、一照一码”整合涉企证照事项45项，企业开办时间压缩到5个工作日，新增各类市场主体32761户。建立投资项目会商调度服务机制，推出472项“最多跑一次”服务。“双随机一公开”市场监管机制进一步健全，全市信用平台实现旗县区全覆盖。全面推行政务服务“十项承诺”，开展企业群众办事“十大烦心事”和百项堵点疏解专项行动，取消34个证明材料，网上办事大厅实现“单点登录、一号查询、一网通办”。包钢入选国家第三批混合所有制改革试点，国有企业“三供一业”、教育医疗机构分离移交工作基本完成。政府机构改革工作有序推进。深度融入“一带一路”建设，建成包头保税物流中心（B型），国家进口肉类指定口岸通过自治区预验收，满都拉口岸过货量完成312.2万吨，增长38%。包头机场航空口岸常年开放列入国家审理计划，国际航站楼实现试运行。组织100多家企业参加首届中国国际进口博览会，成交额居全区首位。北奔重卡宽体自卸车进入国际市场，全市外贸进出口总额完成167.8亿元，增长24.5%，获评全国外贸百强城市、“有色金属材料”和“黑色金属材料”国家外贸转型升级基地。承办呼包鄂第二届市长联席会，112个协同发展项目稳步实施。

（五）城乡环境展现新貌。启动新一轮城市总体规划编制，深入推进“多规合一”，推动“三红一绿一蓝”划定和立法工作，完成518平方公里范围城市开发边界划定。全面开展城市设计和城市“双修”试点工作。持续推进110国道、沼南大道等道路新建改造工程，完成友谊大街、民族西路北延、民主路转盘改造。新都市区、北梁腾空区基础设施水平不断提升，地下管廊、海绵城市、充电桩、停车场等工程加快推进，赛汗塔拉、奥林匹克公园“城市绿肺”功能更加完善。棚改安置房开工11036户，完成年度任务的101%，城镇住房保障家庭租赁补贴完成全年任务的133.5%。出台网约车经营服务管理办法，巡游出租车电召平台建成运营。深化城管执法体制改革，清理整治私搭乱建15.7万平方米，拆除存量违建85.6万平方米。深入实施物业管理提档升级三年行动计划，新增物业达标住宅小区146个，达标率从45%提高到60%，全市301个老旧小区实现准物业管理全覆盖。制定实施乡村振兴战略规划及农村牧区人居环境整治三年行动方案，全力推进村容村貌整治等“七大工程”，478个村庄环境明显改善。国道331线包头边防公路、省道104线希拉穆仁至百灵庙一级公路、省道311线固阳至武川一级公路建成通车，达茂通用机场建成试飞。“四好”农村路建设扎实推进，全年建成农村公路330公里，全市农村公路管理率和行政村通客车率均达到100%。完成偏远农村牧区用电升级改造，行政村光纤网络基本实现全覆盖。土地确权、草牧场登记颁证基本完成，获评全国集体产权制度改革整市推进试点。完成国家重点林业工程58.6万亩，大青山南坡地质环境治理和生态修复成效明显，沿黄湿地得到有效保护，森林覆盖率提高到17.8%，天然草原植被盖度达到35.7%。装备制造园区入选国家绿色园区，北方稀土等4家企业成为国家级绿色工厂，工业能耗智慧监管平台实现试运行，绿色建筑设计建造面积居全区首位，获批国家可再生能源综合应用示范区。

（六）人民群众得到更多实惠。新增城镇就业4.31万人，大学生就业2.5万人，城镇登记失业率控制在3.86%。发放创业担保贷款6.1亿元，新增自治区众创空间7家。社会保险参保扩面4.8万人，发放各类兜底保障资金2.3亿元，企业退休人员养老金、城镇和农村牧区低保标准进一步提高，各项救助保障水平保持全区前列。新建日间照料中心、农村牧区互助幸福院等养老服务机构93个，民政福利园区一期工程、残疾人康复中心基本建成。与全国26个省市实现了跨省异地就医直接结算。成立市旗（县）退役军人服务管理中心，解决了一批涉军群体历史遗留问题。实施教育提升三年行动计划，新改扩建中小学、幼儿园23个，包一中新校区主体完工。公办和普惠性民办幼儿园覆盖率达到82%。学前教育到高等教育贫困学生资助政策实现全覆盖。全面开展校外培训机构专项治理，整改机构357家，依法取缔和申请停办54家。优利康达风电实训基地建成运营，累计输送专业技术人才5000人。全面启动文化产业发展三年行动计划，包头美术馆青山分馆正式运行，乌兰牧骑事业加快发展，电视剧《安居》荣获“飞天奖”优秀电视剧大奖，承办了辽吉黑蒙优秀剧目展演等文化艺术活动。健康城市建设加快推进，市儿童医院一期工程投入使用，新增3个医疗院士工作站。公立医院改革不断深化，获批国家级流动人口基本公共卫生计生服务均等化示范市，实现国家卫生城市“三连冠”。奥林匹克公园“两馆一中心”投入使用，人均体育场地面积提高到2.29平方米，全民健身参与度不断提高，“部校通”足球改革经验向全国推广。警务机制改革初见成效，“三见警”巡逻机制实现常态化，“24小时警局”和公安民警“亮身份、当楼长、保平安、护稳定”活动受到社会广泛好评，禁毒、反恐和“扫黑除恶”专项斗争扎实推进。食药安全形势总体平稳，非洲猪瘟疫情得到有效控制，安全生产大检查、信访矛盾化解攻坚、拖欠农民工工资治理百日攻坚等专项行动深入实施，刑事类警情、安全生产事故、信访批次和人数分别下降62.1%、25.8%、4.6%和17.3%，人民群众安全感和满意度明显提升。全面落实党的民族宗教政策，大力支持国防和军队改革，军民同心、民族团结、宗教和顺、边疆安定的大好局面更加稳固。市侨联荣获全国侨联系统先进集体。审计、统计、人防、档案、气象、地震、残疾人、妇女儿童、红十字等事业持续健康发展。

（七）政府自身建设不断加强。牢固树立“四个意识”，全面落实习近平总书记对内蒙古重要讲话重要指示精神，以实际行动坚决践行“两个维护”。认真落实全面从严治党要求，严格执行中央八项规定及其实施细则精神和自治区、市委配套规定，扎实开展“作风建设年”活动，全面推进党政机关办公用房整改，市本级“三公”经费支出下降18.3%。深入推进法治政府建设，积极开展“七五”普法宣传，修订市政府工作规则，完善市政府党组“三重一大”议事制度，坚持重大事项报告和通报制度。依法执行市人大决议决定，自觉接受市人大法律监督、工作监督和市政协民主监督，全年办理人大代表建议和政协委员提案572件，办复率100%。大力推进诚信政府建设，全面开展拖欠民营企业账款清理工作，政令畅通、诚实守信的社会环境加速构建。

各位代表！过去一年，我们在外部经济环境复杂多变、固定资产投资整体放缓的形势下，深入实施招商引资“市长旗县区长工程”，一批产业转型升级项目基本实现当年开工当年投产，全年固定资产投资总量位居全区首位，实体经济基础不断夯实，工业大市地位进一步巩固。我们在财政增收困难、化债任务艰巨的情况下，集中财力保民生、保重点，民生支出287亿元，解决了生态环境领域一批长期想解决而没能解决的历史遗留问题，全市上下齐心协力，成功抗击了历史罕见的重大洪灾，妥善安置了房屋受损群众。我们在经济转型升级阵痛、深层次矛盾集中显现的环境下，更加注重打基础、利长远，编制了12条重点产业链图和高质量发展等系列战略规划和实施方案，实施了一批关键技术攻关，培育了74户国家级高新技术企业，累计达到160家，全力推动经济向高质量发展迈进。我们在规范政府债务管理、压减政府投资项目的基础上，聚焦强优势补短板，通过企地融合、深化改革等一系列措施，坚定了企业发展信心，汇聚起了“大包头”共识，形成了共谋发展的良好氛围，288万包头人民对未来的发展充满信心。

困难中的转机来之不易，压力下的收获弥足珍贵。这是以习近平同志为核心的党中央坚强领导、亲切关怀的结果，是自治区党委、政府正确领导、关心支持和市委统揽全局、科学决策的结果，是市人大、市政协大力支持、有效监督的结果，是各地区各部门和广大干部同心同德、攻坚克难的结果，是全市人民负重自强、苦干实干的结果。在此，我代表市人民政府，向全市各族人民，向各民主党派、工商联、人民团体、无党派人士，向驻包部队、武警官兵和驻包单位，向所有关心和支持包头发展的企业家以及社会各界人士，致以崇高的敬意和衷心的感谢！

在总结成绩的同时，我们也清醒地认识到，包头发展仍处在爬坡过坎的艰难时期，还面临着不少困难和挑战。产业“四多四少”问题还很突出，战略性新兴产业尚未形成关键支撑，现代服务业发展与城市地位不相匹配。国有企业、科技创新、稀土产业等比较优势尚未形成集聚效应，行业企业条块分离的体制机制障碍需要进一步破除。固定资产投资指标没有达到预期，扭转低位运行态势、扩大精准有效投资还需付出更大努力。营商环境有待进一步改善，一些改革举措和政策落实不到位，民营经济发展不充分。项目投产达效率不高，税收增长动力不足，政府化债任务艰巨，财政收支矛盾突出。节能减排任务依然很重，工业集中区、城郊结合部空气质量亟待改善。民生领域尚有不少短板，距离人民群众的期盼还有差距。部分干部危机意识和紧迫感不强，思想观念、创新意识、担当精神还不适应新形势发展需要。对此，我们要增强忧患意识，采取有力措施，认真加以解决。

二、2019年工作安排

今年是新中国成立70周年，是决胜全面建成小康社会的关键之年。政府工作的总体要求是：坚持以习近平新时代中国特色社会主义思想为指导，全面贯彻党的十九大和十九届二中、三中全会精神和习近平总书记参加十三届全国人大一次会议内蒙古代表团审议时重要讲话精神，认真落实中央、自治区和市委的各项决策部署，统筹推进“五位一体”总体布局，协调推进“四个全面”战略布局，坚持稳中求进工作总基调，坚持新发展理念，坚持推动高质量发展，坚持以供给侧结构性改革为主线，坚持深化市场化改革、扩大高水平开放，加快建设现代化经济体系，继续打好三大攻坚战，着力激发微观主体活力，统筹推进稳增长、促改革、调结构、惠民生、防风险工作，保持经济运行在合理区间，进一步稳就业、稳金融、稳外贸、稳外资、稳投资、稳预期，提振市场信心，增强人民群众获得感、幸福感、安全感，保持经济持续健康发展和社会大局稳定，为全面建成小康社会收官打下决定性基础，以优异成绩庆祝中华人民共和国成立70周年。

今年主要发展预期目标是：地区生产总值增长6.5%左右，规模以上工业增加值增长9%左右，社会消费品零售总额增长7%左右，一般公共预算收入增长6%左右，城乡居民人均可支配收入分别增长7%左右和8%左右。城镇登记失业率控制在3.9%以内。全面完成自治区下达的节能减排约束性指标。

做好今年的政府工作，要重点把握好以下几个方面：

一是始终坚持发展第一要务不动摇。发展是解决一切问题的基础和关键，发展必须是科学发展和高质量发展。包头的发展正处于转型升级、体制再造、重新出发的关键阶段，急需解决质量不优、效率不高、动力不足等难题。我们的一切工作要围绕发展这个主题，紧紧扭住经济建设这个中心，以项目建设和有效投资带动质量变革，以资源要素优化配置促进效率变革，以改革创新推进动力变革，用发展的办法解决发展中的问题，以经济高质量发展引领各项事业提档升级、争创一流。

二是坚定不移推动企地融合发展。企地融合是厚植国有经济发展优势、带动民营经济发展壮大的重要举措。国有企业集聚是我们最大的优势，民营经济振兴是我们最大的潜力，我们要始终坚持“两个毫不动摇”，牢固树立“大包头”“一盘棋”理念，同心同向推动驻市企业、科研院校、金融通信等优势资源同地方经济深度融合，汇聚起企地双方融合共赢的强大合力，构建起国有民营经济相得益彰的发展格局。

三是践行以人民为中心的发展思想。带领群众不断创造美好生活，是我们矢志不渝的奋斗目标。尽管当前可用财力十分有限，但是我们将坚持尽力而为、量力而行，确保涉及群众切身利益的民生保障力度不减、标准不降，多做雪中送炭、急人之困的实事，一步一个脚印把民生工作做实做好，努力让广大群众在迈向全面建成小康社会的路上更加踏实、更有保障、更可持续。

四是弘扬担当务实、争创一流的精神。包头六十多年的艰苦创业历程启示我们，走出困境，踏上高质量发展快车道，没有灵丹妙药，没有捷径可走，唯有凝心聚力、埋头苦干。我们要激励和引导广大干部群众以敢为人先的志向、凤凰涅槃的决心、务实担当的作风，不为困难所低头，多为发展想办法，心往一处想、劲往一处使，切实凝聚起人人共奋进、人人共担当的磅礴力量，重振包头雄风，再铸新的辉煌。

2019年重点做好以下几个方面工作。

（一）突出强化创新引领，汇聚高质量发展强大动能。创新是推动高质量发展的动力引擎，我们要深入实施创新驱动战略，加速培育新动能。

以体制创新推动企地融合发展。完善企地融合发展政策，优化提升六大平台功能，引导支持企地双方资源要素有序开放、双向流动，着力提升全要素生产率。加强企地协同机制建设，建立以政府为主导的产业链“链长制”和以国有企业为主导的产业集群“群长制”，着力破除制约企地融合发展的体制机制障碍，推动产业链条垂直整合、配套企业集聚共生。以国有企业混合所有制改革为契机，扩大民营企业参与融合份额，全力实施北方稀土高效稀土永磁产业化等企地融合项目，共同谋划一批高端产业项目，不断提升大型国有企业地方配套比例，加快形成优势产业集群。建立健全政企学研协同创新机制，支持各类研发机构参与国有企业技术改造和关键核心技术攻关，加快构筑国有企业和地方经济共赢共享新格局。驻市国有企业要有担当精神，进一步发挥支撑引领作用，在转型升级中勇当排头兵、顶梁柱。

以服务创新促进民营经济发展。认真落实支持民营经济发展的六项措施。全面落实国家更大规模的减税降费政策，采取支持民营企业参与电力多边交易、推广多式联运、整治涉企收费等多种方式，着力减轻企业成本和税费负担。设立中小企业可持续发展基金，搭建“包头市融资服务平台”，推行“信贷白名单”制度，扩大“助保金”贷款覆盖面和资金池，加快破解民营企业融资瓶颈。全面开展市场准入限制专项清理行动，常态公布面向民间资本项目清单，落实现有各类企业新上项目或重大技术改造享受招商引资同等政策，着力营造公平公正的市场环境。认真落实“弘扬企业家精神十条”，健全企业家参加涉企政策制定机制，依法保护企业家合法权益。加大各类政策措施落实和兑现力度，让市场主体享受到更多更实的政策红利。着力构建亲清政商关系，全力营造企业安心放心发展的社会环境。全面做好“抓大、扶小、扭亏、育新、延链”五篇文章。多措并举帮助龙头企业稳市场、稳订单、稳生产，支持民营龙头企业做大做强，鼓励创建商标品牌。加大中小企业培育支持力度，培育专精特新中小企业50户。开展领导干部包联服务百户重点民营企业专项行动，积极帮助企业解决实际问题。推动更多科技创新资源向民营企业开放共享，引导民营企业围绕重点产业链布局项目，利用技术创新和规模效应形成竞争新优势。深入实施质量提升行动，弘扬劳模精神、工匠精神，推进全国质量强市示范城市建设，打造更多“百年老店”。民营企业要坚定发展信心，踏踏实实搞实业、专心专注做主业，努力在高质量发展的征程中再立新功。

以科技创新助推优势产业发展。加快推进国家军民融合创新示范区、国家稀土技术创新中心等平台建设，全力推动中科院包头稀土功能材料研究院尽快落地，培育建设20家自治区级以上创新平台。支持企业加大科技研发投入、创建国家级创新中心，新增高新技术企业50家、创新型企业50家、民营科技创新引领型企业50家、科技小巨人企业20家、军民融合科技示范企业10家，组织实施重点科技项目50项以上。加快推动稀土新材料、铝镁深加工、煤制烯烃深加工中试基地建设，支持中科院包头科技产业园、上海交大包头新材料产业园、包头稀土研究院等5个科技成果转化示范基地建设。支持50家研发机构带头推进关键核心技术攻关和科技成果转化，推动稀土陶瓷、高性能稀土铝钛合金等15项成果落地。研究制定新旧动能转换先行区科技创新若干意见政策，建立多元投入的科技金融体系。积极参与各类标准体系建设，进一步增强包头产品的核心竞争力和市场话语权。加大对重大装备和关键产品的支持力度，把首台首套政策落到实处。加快中关村信息谷创新中心建设，强化脉点科技包头频道功能，加强“高精尖缺”人才引进和知识产权保护运用，赋予研发机构和科研人员更大自主权。切实发挥好“科技十条”“人才十条”及产业基金、科技创新基金等政策的综合效益，打造充满活力、更具竞争力的创新生态系统。

以管理创新优化园区投资环境。调整优化产业布局，因地制宜定位园区功能，推行“飞地经济”模式，突出园区特色，避免同质竞争，促进产业集约集聚集群发展。完善工业园区考核办法，建立亩均综合效益评价机制，开展低效用地盘活再利用行动，依法整治圈而未建、圈多用少、占而不用等用地行为，提高单位面积投入产出强度。加强园区基础设施建设，实施标准化厂房等32个基础设施项目，创新公共服务供给体制和园区投融资体制，提升园区承载能力。进一步理顺工业园区管理体系，构建服务管理高效、推进协调便捷的体制机制。

以金融创新促进实体经济发展。丰富金融产品和服务模式，设立区域产权和金融资产交易平台，加快推动内蒙古金融租赁公司落户我市。发展多层次资本市场，支持明拓集团、天和磁材加快在主板上市，鼓励符合条件的企业发行债券，大力发展股权交易，扩大直接融资比例。优化包钢等驻包大企业债务结构，加快市场化、法制化债转股进程。坚决打好防范化解重大风险攻坚战，抓住中央较大幅度增加地方政府专项债券规模的政策机遇，积极争取新增债券限额和专项债券额度。树立经营城市理念，加快平台公司实体化转型，鼓励民间资本参与政府和社会资本合作项目建设。围绕“坚定、可控、有序、适度”的要求加强政府债务特别是隐性债务管控，严控增量、化解存量，确保完成年度债务化解任务。强化金融风险预警监测，加大非法集资、企业信用债违约等行为的打击力度，推动建立金融法庭，严防各类金融风险。

（二）调整优化产业结构，加快转变经济发展方式。产业是推动高质量发展的重要支撑，我们要坚持以项目引领带动产业转型，实施624个投资亿元以上重大项目，完成投资1000亿元以上，年内竣工200个以上，不断增强实体经济发展后劲。

改造提升传统产业。优化钢铁企业生产工艺，提高产品技术含量和质量效益，加快推进明拓80万吨稀土现代铁素体不锈钢、威丰冷轧取向硅钢三期等项目建设，积极发展电力用特种钢、高铁重轨、汽车用高品质钢等高端产品。着力推进隆华30万吨铝合金材料、华云轻合金材料三期等项目，积极发展新型铝合金、高品质铝铸件、铝铜镁压延加工等下游产品。制定出台消费品工业振兴实施意见，加快东宝生物、英华融泰等一批数字智能化车间建设，培育壮大羊绒纺织、粮油、乳肉、日化等加工产业，推动消费品工业振兴发展。

培育壮大新兴产业。加快发展清洁能源产业。启动国家级可再生能源综合应用示范区和5个工业园区配售电改革试点建设，实施好采煤沉陷区光伏基地、包头北千万千瓦级大型现代风电基地等项目，加快山北新能源至山南工业园区清洁能源消纳输送通道和特高压电力外送通道建设，提高能源综合利用效率和供应保障能力。加快发展高端装备制造业。实施双马微涡轮能源动力装置、中铁盾构机、比亚迪动力电池等现代装备制造项目，加快推进国家级应急救援装备制造基地建设，培育壮大环保设备、机器人制造、轨道交通设备和新能源汽车及零部件等高端装备制造业。加快发展军民融合产业。设立军民融合科技发展专项，加快推动泳池式低温供热堆、一机集团传动基地等12个军民融合项目，支持高品质钢、稀土功能等新材料，高效电机、动力电池等关键部件，特种车辆、军民两用装备等产品参与军工装备制造，带动形成优势产业集群。加快发展智能制造产业。大力实施智能产品研发创新、生产过程智能化升级、智能化服务和云平台建设四个专项行动，培育一批智能制造重点示范区和骨干企业，推动一机集团智能仿真、北奔“北斗鑫”智能车辆等系统技术延伸和突破升级，扩展内蒙古网络协同制造云平台服务领域，全力创建国家级智能制造和工业互联网示范试点。

优先发展稀土新材料及应用产业。全力推动全国稀土新材料、高端应用技术研发和产业化示范基地建设，引进储氢电池等院士工作站，攻克一批稀土新材料领域的共性关键技术和前沿技术。深入实施“稀土+”战略，以稀土合金、磁材研发生产为突破口，助推稀土新材料和永磁电机、伺服电机、新能源汽车动力电池等稀土下游终端产品发展，带动制造业全面转型升级，打造稀土产业“升级版”。加快稀土新材料产业园区20平方公里核心区建设，实施好玉立稀土材料、中晶永磁电机等40个项目，新增入驻企业15家，推动稀土初级加工项目向山北地区有序转移。建立完善稀土新材料技术评价指标体系，支持包头稀土研究院创建国家级稀土新材料测试评价中心，推动包头稀土产品交易所扩大交易范围、完善交易体系，加快打造世界稀土产业之都。

大力发展现代服务业。制定出台服务业发展三年行动计划，加快发展十大生活性服务业和十大生产性服务业，积极推进“8个5”品牌示范企业创建活动。提档升级生活性服务业。加强新都市区周边文化体育场馆综合利用，推动万科、中东奥特莱斯等商业综合体建设，丰富商圈业态，打造辐射呼包鄂榆区域高端化个性化消费中心。推进五当召5A级景区等景点创建，加快春坤山、森林春天和小白河文旅综合体等31个项目建设，支持石拐区、土右旗、达茂旗、固阳县创建全域旅游示范区。发展工业和军事旅游、红色旅游、黄河旅游、特色小镇旅游，办好游牧文化旅游节等品牌活动。扶持壮大文化企业，积极培育文化创意、数字出版、网络视听等新兴业态。大力发展养老产业，加快推动市福利园区庭院式养老等项目建设，新建社区日间照料中心、农村牧区互助幸福院等各类养老服务机构100个，新增2000张养老床位，力争90%以上养老机构实现医养结合。积极发展健康、住宿餐饮、家庭服务、教育培训、体育服务和法律服务等生活性服务业，更好满足群众多样化消费需求。培育壮大生产性服务业。出台工业设计产业创新发展的政策措施，推进稀宝博为等工业设计平台建设，培育认定一批工业设计中心。推动钢铁建材、装备制造、铁路综合等物流园区提档升级，支持“无车承运”“多式联运”等物流模式创新试点建设，发展冷链物流、城乡配送、智慧物流等新业态。做大做强钢铁、煤炭等电商交易平台，推进大红堂跨境电商、苏宁小店等项目建设，创建国家电子商务示范基地。办好办精稀土产业国际论坛、牛羊肉产业大会等特色展会，推动会展业向专业化、国际化、品牌化发展。强化数据信息资源开发应用，加快内蒙古软件园、包头云计算中心等项目建设，引进一批大数据知名企业，加速催生新产业新业态新模式。大力发展节能环保、检验检测、商务咨询、外包售后、人力资源等生产性服务业，更好满足产业升级需求。扎实做好第四次全国经济普查工作，确保做实数据、摸清家底。

发展“高精强特优”现代农牧业。坚持质量兴农、绿色兴农、品牌强农，完善5个现代农牧业示范园区功能，改造提升3个大型食品加工园区，力争入园企业突破200家，农畜产品加工转化率达到68%以上。加快农牧业科研示范等基地建设，承接设施绿色防控等20项科研项目，推动设施农业提档升级。大力发展都市型现代农业，因地制宜发展特色种养、乡村旅游、家庭农场等新业态新模式，按照规模化特色化标准化要求建设休闲农牧业示范基地和田园综合体10个，让小农牧户更多融入现代农牧业发展大格局。扩大土地流转规模，打造农牧业标准化生产基地60个。开展“中国好粮油”包头行动，全面落实粮食安全盟市长责任制。增加绿色优质农畜产品供给，新增“三品一标”认证产品40个，打造黄芪、肉羊等特色优势产品“包字号”名片。健全完善农畜产品质量安全追溯体系，实现土右旗、达茂旗规模以上重点生产经营主体追溯全覆盖。

（三）持续深化改革开放，充分激发市场活力动力。改革开放是推动高质量发展的关键一招，我们要以壮士断腕的决心深化改革，以包容共赢的理念扩大开放，着力破解制约发展的瓶颈。

深化重点领域改革。高质量完成机构改革任务，进一步理顺政府部门职责关系。深化财政体制改革，加强全口径预算编制和预算资金绩效管理，提高专项资金使用效率。推进国资国企改革，实现经营性国有资产集中统一监管。加强国有企业、行政事业单位资产管理，完善国有资本经营预算制度，推动市属国有经济整合重组，加快实现从管企业向管资本转变。理顺国有企业教育医疗机构移交后的管理体系。深化集体林权制度改革，鼓励各类社会主体依法依规参与林权流转，不断完善生态文明制度体系。

全面优化营商环境。对标国际标准建立包头营商环境目标任务和评价指标体系，将营商环境优化成效列入年度考核内容，加速构建责任明确、运转高效、监督有力的工作机制。深化“放管服”改革，加快推动“照后减证”，着力破解“准入不准营”的问题。大力推行联合审批、联合验收、联合踏勘、联合测绘、多图联审和区域评估，加快投资项目“告知承诺制+信用监管”改革，进一步提高审批效率。以机构改革为契机，进一步优化环保、消防、税务、市场监管等执法方式，清理规范行政处罚事项，强化事中事后监管，严肃整治损害营商环境行为。

全面推进“互联网+金融+政务服务”，建立完善跨部门、跨地区的信息共享平台和基础数据库，打通与国家、自治区互联互通的政务服务通道，实现审批服务事项“应上尽上、全程在线”。启动市县乡村四级政务服务标准化建设，加快全市各类政务服务热线整合，推动更多行政审批和公共服务事项进驻市民大厅，实现“一网、一门、一次”改革目标。

提高招商引资实效。持续实施招商引资“市长旗县区长工程”，引导各地区细化实化重点产业链图，围绕12大重点产业和新兴业态培育，有针对性地谋划引进产业链上的关键企业、核心企业、上下游配套企业。探索建立重大招商项目特殊政策支持机制，制定特色化、差异化招商实施方案，组建招商引资专家顾问团队和专业招商队伍，实施精准招商、以商招商，更多引进科技含量高、投资体量大、拉动作用强、财税贡献多的产业项目，力争引进国内（区外）到位资金增长10%以上。

扩大对外开放格局。完善满都拉口岸功能，加快推动满都拉铁路口岸申报工作，推动北方工业跨境物流园区落地实施，过货量突破340万吨，增长10%。开工建设包银高铁，积极推动包西高铁前期工作。启动运营国家进口肉类指定口岸、包头保税物流中心（B型），顺利完成包头机场航空口岸国家验收。积极融入“一带一路”和“中蒙俄经济走廊”，组织参加第十五届中俄蒙工商论坛等国际交流活动，支持企业走出去建设生产加工基地、营销网络和配送体系，推动神华聚烯烃、北奔重卡等优势产品出口。推动呼包鄂协同发展，着力推进基础设施互联互通、产业链条顺畅完备、产业发展分工协作、特色产业集群发展、科技创新联合攻关、公共服务共建共享，加快创建呼包鄂国家自主创新示范区。积极融入京津冀协同发展，力争在技术创新合作、产业转移承接等领域取得突破。

（四）统筹城乡协调发展，系统提升城市品质内涵。城市是推动高质量发展的重要载体。我们要尊重城市发展规律，提高城市承载能力，传承城市人文精神，真正把包头建设成为一座“来了就不想走”的城市。

不断提升城市功能品质。秉承建市以来形成的良好规划基础，高起点高质量做好新一轮城市总体规划编制，强化规划刚性约束，坚决守好“三红一绿一蓝”五条线。全面完成城市设计、城市“双修”试点工作。打通赛汗路南段、站前路2条断头路，做好城市快速路规划建设前期工作，实现110国道、沼南大道全线通车，形成两环五横八纵路网体系。新建5处人行过街天桥和地下人行通道，实施8条道路拓宽改造和一批路口渠化工程，增加1.5万个公共停车位，新建改造6处公共停车场，有效解决道路拥堵、停车难问题。加快推动6处老旧管网、15处雨污管网分流改造和主城区36处积水点治理，排除城市内涝隐患，实现雨污分流。实施集中供热环城北干线续建工程，新增供热面积300万平方米。全面推进东河区东部区给排水一体化工程。新增管道直饮水设施覆盖人口6万人。加快创建国家生态园林城市，改造森林公园，新建一批中小型公共绿地，基本实现主城区“300米见绿、500米见园”全覆盖。完成自治区下达的棚户区改造任务，改造国有企业移交老旧小区71个、550万平方米，新增物业达标住宅小区147个，达标率提高到75%。在增加租赁住房供应、开展房地产市场秩序整治等5个方面下功夫，推动房地产市场平稳健康运行。健全城市精细化治理体系，统筹推进平安包头、智慧教育等应用。改造提升12条城市街景，治理12条街巷，确保新建违法建设管控查处率达到100%，建成区存量违法建设清理比例达到90%以上。加强扬尘、油烟、尾气等污染管控，新建改造105个城市公厕，持续推进城市亮化和节能改造工程，进一步提升城市宜居水平。

着力优化城乡空间布局。积极推进固阳至白云、东河至固阳和黄河大桥通道前期工作，加快北绕城一级公路建设，推动省道315线托县至东河主体完工，确保G6高速青山互通工程、国道210线满都拉口岸至白云鄂博、国道335线黄家滩至百灵庙全线建成通车，完成旅游公路收尾工程，建成固阳、石拐通用机场，加速构建内联外畅的现代交通体系。高标准推进新都市区和北梁腾空区建设，进一步完善基础设施和公共服务网络。鼓励各地区因地制宜建设资源加工、商贸物流等特色小镇，促进城区与城镇协调发展。支持石拐区发展大数据、共享经济等新兴业态，加速融入主城区。支持白云矿区加强多金属资源综合利用和尾矿综合开发利用，打造稀土原材料和尾矿综合利用基地。支持土右旗壮大现代物流、清洁能源产业，加快建设新型中等城市。支持达茂旗发展风光电新能源产业，打造可再生能源综合应用示范区及就地消纳产业链。支持固阳县加快构建铝深加工、石墨烯等支撑产业体系，巩固脱贫攻坚成果。

深入实施乡村振兴战略。健全乡村振兴战略财政投入保障制度，加大涉农涉牧资金统筹整合力度，创新金融支农支牧产品服务，撬动更多社会资本投向农村牧区。扎实开展以“三清一改”为重点的村庄清洁行动，突出抓好垃圾污水治理、厕所革命、村容村貌提升等专项行动，确保建制村环境综合整治比例达到80%以上。实施农村牧区基础设施提升工程，新建改建208公里农村公路，扩大行政村4G信号覆盖范围，管好用好已建成的各类设施。推进农村土地“三权分置”改革，完成全国集体产权制度改革整市推进试点任务，盘活农村牧区“沉睡资本”，发展壮大村集体经济，努力让农牧民有更稳定的收入。健全完善农民工返乡创业政策，扶持培养一批农业职业经理人和职业农民，吸引支持企业家等社会人士投身乡村振兴事业。传承优秀乡土文化，广泛开展“五好家庭”等评选表彰活动，提升文明素质、弘扬时代新风。

巩固提升城市文化内涵。深入践行社会主义核心价值观，弘扬艰苦奋斗的创业精神，厚植包容开放的城市特质，擦亮全国文明城市“金字招牌”，传承好城市文化，守好精神家园。启动内蒙古工业历史博物馆建设，完善基层公共文化服务设施，不断提升城乡公共文化设施标准化、服务均等化水平。办好鹿城文化艺术节、交响音乐季等品牌文化活动，组织文艺院团、乌兰牧骑开展“扎根基层、服务人民”等主题实践活动，举办艺术惠民演出600场以上。围绕新中国成立70周年主题，挖掘深厚多元的文化底蕴，打造歌剧《双翼神马》、话剧《为正义辩护》等一批精品力作，演绎新时代包头精彩篇章。加大文物认定保护力度，深入推进“草原神鹰”工程，做好五当召、美岱召文物保护修缮工作，打造好北梁“老包头记忆”等特色街区，延续城市“文脉”，留住城市“基因”。

（五）加强生态环境治理，全面提升生态文明水平。生态建设是推动高质量发展的题中之义。我们要坚决践行绿色发展理念，持之以恒推进生态建设和污染治理，为构筑祖国北疆万里绿色长城贡献包头力量。

打好污染防治攻坚战。强化重点领域污染综合治理，如期完成中央环保督察及“回头看”反馈意见整改工作。持续推进“治气、减煤、降尘、控车、严管”5项措施，推动工业重点企业开展深度治理，大力开展禁燃区煤改气、煤改电工程，加大高污染车辆、“三烧三尘”管控力度，推动“国三”及以下排放标准柴油货车淘汰更新，持续提高空气质量优良天数比例，坚决打赢蓝天保卫战。加快实施加通污水处理厂改造、希望铝业园区再生水管线、二道沙河生态治理等工程，持续推进东华热电等工业企业废水资源化利用，力争生活污水集中处理率达到95%，再生水利用率达到22%以上。加强集中式饮用水水源地保护，完成昆区、东河区地下水超采治理，封停地下水源井60眼，进一步提升饮水安全保障水平。扎实开展土壤污染防治工作，推动农药、化肥、地膜减量使用，加大重金属污染防治力度，完成90%以上地下油罐防渗改造任务。全面强化固废危废综合安全处置，支持工业固废利用特色示范基地建设。加快推动生态环境保护立法工作。建立完善环境监管“地空天”系统，持续开展“环保利剑”系列专项行动，改进生态环境执法方式，与企业共同制定环境治理解决方案，坚守阵地，巩固成果。

扎实推进生态建设。统筹山水林田湖草治理，实施林业重点工程40万亩以上，打造大青山南坡、沿黄河2条绿色走廊和土右旗东线、昆河西线2个绿化带，逐步实现“东西南北、生态环城”的绿化格局。全力推行“山长制”管理模式，完成大青山南坡矿山地质环境及生态恢复治理任务和7个绿色矿山建设工作。深入落实河长制、湖长制，实施好城市水生态综合治理工程和南海湿地修复项目。落实草原生态补奖政策，巩固围封禁牧、退耕还林还草成果，持续改善草原生态。

促进绿色低碳发展。强化能源和水资源消耗、建设用地等总量和强度“双控”行动，建成工业能耗智慧监管平台，严把新建项目能耗准入门槛。加快推动生活垃圾资源化分选、餐厨垃圾处置二期项目建设，日处理能力达到200吨，进一步提升生活垃圾无害化处理率。做大做强国家“城市矿产”示范基地，提升资源循环利用水平。大力推广绿色建筑、绿色交通、绿色消费、绿色能源，倡导全社会形成绿色环保的生产生活方式。

（六）着力保障改善民生，不断增强人民群众获得感幸福感安全感。增进民生福祉是推动高质量发展的根本目的。我们要完善制度、守住底线，多解难题、多办实事，努力让群众生活更加幸福美满。

打好打赢精准脱贫攻坚战。建立健全与扶贫任务相适应的财政投入保障机制，严格执行贫困退出标准和程序，进一步增强精准度和实效性，确保剩余1688名建档立卡贫困人口全部脱贫。按照“六个精准”要求，聚焦“两不愁、三保障”，细化强化“五个一批”扶贫举措，因地制宜推进特色产业扶贫，把更多贫困户吸附在产业链上。做好易地扶贫搬迁村产业配套和基础设施、公共服务配套工作，确保“搬得出、稳得住、能致富”。不断完善城区优质学校与农牧区中小学、幼儿园对口帮扶共建捆绑考核机制。深入实施健康扶贫“三个一批”行动计划，防止因病致贫、因病返贫。健全贫困人口低保、基本养老保险等政策兜底保障体系，做到应保尽保。统筹做好政策培训、技术指导、典型带动、模式创新等工作，强化智志双扶，激发贫困人口内生动力和自我发展能力。探索建立略高于建档立卡贫困户群体的常态化帮扶机制，防止相对贫困群体成为新增贫困人口。持续加大扶贫领域作风问题专项治理力度，举一反三抓好中央扶贫专项巡视反馈问题的整改落实，确保脱贫攻坚成果经得起历史检验。

提升就业和社会保障水平。突出产业带动就业，通过产业转型、培育新经济新业态新模式创造更多大学生就业岗位，扎实做好失业人员再就业、复员军人转业安置和农民工等特殊群体就业工作，全年新增城镇就业4.2万人以上。深入推进“创业包头”三年行动计划，完善创业园孵化基地服务体系和多层次、全覆盖培训体系，扩大创业担保贷款覆盖面，为更多创业者搭建实现梦想的舞台。实施高技能人才“聚匠工程”和“三年引育工程”，培养90名国际认证高技能人才，加快包头高技能人才公共实训基地建设。优化社保基金收支管理，坚决查处骗保行为，把老百姓的保命钱、救命钱管好。落实社会保障提标扩面政策，提高社会保险待遇水平和弱势群体保障标准，建设民政福利园区二期工程，努力让困难群众的基本生活更有保障。

加快建设教育强市。优化教育资源供给，新改扩建中小学11所、幼儿园6所。建立健全多元化投入和多形式办园体制，加快补齐学前教育短板。持续深化招生制度改革，着力推行学区化管理和义务教育学校“多校划片”招生试点，逐步解决“城区挤”“农区弱”“择校热”“大班额”等问题。改进普通高中育人模式，不断提高名优高校升学比例，努力让每个孩子都能享有公平而有质量的教育。加快改进职业教育模式，积极推动校际之间资源共享，大力培养实用技术技能人才。推动校地融合，支持内蒙古科技大学等高等院校围绕全市重点产业开展技术研发和成果转化。加强师德师风建设，深入实施“领航校长”“卓越教师”培养计划和乡村教师支持计划，完善教师引进补充政策，全面促进各级各类教育高质量发展。

提升人民健康水平。优化医疗资源布局，积极推进包医国际医院、市第八医院医养联合体等项目，发挥医疗院士工作站和博士工作站作用，促进全国优质医疗资源向我市延伸。持续深化公立医院综合改革，积极推进医联体建设，进一步扩大异地就医直接结算和定点医疗机构覆盖面，提升心理咨询、健康教育等现代医疗服务水平，方便群众看病就医。启动包头市全民健康信息平台建设，完善便民惠民信息系统服务功能。抓好蒙中医药传承创新，振兴蒙中医药事业发展。广泛开展全民健身活动，举办首届包头国际马拉松、全民健身大会等体育赛事，投放60套全民健身路径，积极推动更多的公共体育场馆设施向群众免费开放。全力推动足球改革发展试点建设，办好全国青少年足球夏令营等活动。

维护社会和谐稳定。加强城乡社区服务能力建设，精简规范社区工作，让社区更好地为群众服务。完善治理拖欠农牧民工工资问题长效机制，让每一位农牧民工的辛勤付出得到应有回报。强化安全生产责任制，实行安全生产网格化管理，严格落实企业主体责任、行业监管责任、属地管理责任，用严密的责任体系坚决遏制重大安全事故发生。全面加强应急管理，提高防灾减灾和应急救援能力。开展校园及周边、农贸市场、食品生产加工小作坊专项整治，加大药品、医疗器械等领域监管力度，加快创建国家食品安全城市。严格落实定期接访、带案下访和领导带头包案等制度，持续推进信访积案化解，及时有效解决群众合法诉求。深化“平安包头”建设，严格执行“三见警”机制，完善“24小时警局”平台功能，全面加强涉稳隐患排查和公共安全管理，严厉打击各类违法犯罪，全力打好“扫黑除恶”专项斗争和反恐、禁毒硬仗。扎实做好民族团结进步和宗教工作，支持国防和军队改革发展建设，争创双拥模范城全国“九连冠”、自治区“十连冠”。

（七）全面加强自身建设，全力打造现代服务型政府。干部队伍是推动高质量发展的中坚力量。我们将始终坚持对党绝对忠诚的政治品格、勤政务实的工作作风，尽职尽责，担当作为，着力提升政府行政效能和服务水平。

一是坚持党的领导。树牢“四个意识”，坚定“四个自信”，做到“两个维护”，始终在思想上政治上行动上同以习近平同志为核心的党中央保持高度一致。提高政治站位、增强政治担当，坚决贯彻落实习近平总书记对内蒙古重要讲话重要指示精神，全面推动中央、自治区和市委的各项决策部署落地生根、开花结果，确保全市改革发展各项事业始终沿着正确的方向前行。

二是严格依法行政。认真执行市人大及其常委会的决议决定，自觉接受市人大的法律监督和工作监督，主动接受市政协民主监督，高质量办理建议提案。广泛听取各民主党派、工商联、无党派人士和人民团体意见，自觉接受司法监督、群众监督、舆论监督。全面强化政务公开，切实保障人民群众的知情权、参与权、表达权、监督权。深入推进法治政府建设，坚持依法科学民主决策，认真落实“三重一大”议事制度，运用法治思维提升依法履职能力，确保政府工作始终在法治轨道上运行。

三是强化实干担当。营造崇尚实干的良好氛围，用行动诠释忠诚，用责任书写担当，始终保持干事创业的激情和攻坚克难的韧劲。弘扬苦干实干的优良作风，细化任务书、列出时间表、绘出施工图、明确责任人，确保完成全年目标任务。建立真抓实干的管理机制，构建科学有效的考核机制，完善正向激励和容错纠错机制，让广大公务人员想干事、敢干事、能干事、干成事。

四是坚持从严治政。严格落实全面从严治党主体责任，坚决把纪律和规矩挺在前面，着力营造良好的政治生态。深入贯彻中央八项规定及其实施细则精神和自治区、市委配套规定，驰而不息纠正“四风”。强化重点领域和关键环节监督，加强公共资金、国有资产、公共资源交易等领域廉政风险防控，抓早抓小、防微杜渐。树立过紧日子思想，严格控制“三公”经费支出，压减一般性支出9%以上，把有限的资金用到民生最需要的地方。整肃庸政懒政怠政行为，坚决治理政务失信行为，不断提升政府执行力落实力公信力。

各位代表！征程万里风正劲，重任千钧再扬鞭。让我们更加紧密地团结在以习近平同志为核心的党中央周围，在市委的坚强领导下，不忘初心、牢记使命，以永不懈怠的精神状态和一往无前的奋斗姿态，在新时代展现新作为、创造新业绩，用人民满意的成绩庆祝中华人民共和国成立70周年！
